# Volumetric Brain Morphometry Changes in Patients with Obstructive Sleep Apnea Syndrome: Effects of CPAP Treatment and Literature Review

**DOI:** 10.3389/fneur.2014.00058

**Published:** 2014-04-29

**Authors:** Nelly T. Huynh, Olga Prilipko, Clete A. Kushida, Christian Guilleminault

**Affiliations:** ^1^Stanford Centre for Sleep Sciences and Medicine, Stanford University, Redwood City, CA, USA; ^2^Faculty of Dental Medicine, University of Montreal, Montreal, QC, Canada

**Keywords:** obstructive sleep apnea syndrome, volumetric brain morphology, functional magnetic resonance imaging, continuous positive airway pressure, gray matter volume, white matter volume, nocturnal hypoxemia

## Abstract

**Introduction:** Obstructive sleep apnea syndrome (OSAS) is a frequent breathing disorder occurring during sleep that is characterized by recurrent hypoxic episodes and sleep fragmentation. It remains unclear whether OSAS leads to structural brain changes, and if so, in which brain regions. Brain region-specific gray and white matter volume (GMV and WMV) changes can be measured with voxel-based morphometry (VBM). The aims of this study were to use VBM to analyze GMV and WMV in untreated OSAS patients compared to healthy controls (HC); examine the impact of OSAS-related variables (nocturnal hypoxemia duration and sleep fragmentation index) on GMV and WMV; and assess the effects of therapeutic vs. sham continuous positive airway pressure (CPAP) treatment. We discuss our results in light of previous findings and provide a comprehensive literature review.

**Methods:** Twenty-seven treatment-naïve male patients with moderate to severe OSAS and seven healthy age- and education-matched HC were recruited. After a baseline fMRI scan, patients randomly received either active (therapeutic, *n* = 14) or sham (subtherapeutic, *n* = 13) nasal CPAP treatment for 2 months.

**Results:** Significant negative correlations were observed between nocturnal hypoxemia duration and GMV in bilateral lateral temporal regions. No differences in GMV or WMV were found between OSAS patients and HC, and no differences between CPAP vs. sham CPAP treatment effects in OSAS patients.

**Conclusion:** It appears that considering VBM GMV changes there is little difference between OSAS patients and HC. The largest VBM study to date indicates structural changes in the lateral aspect of the temporal lobe, which also showed a significant negative correlation with nocturnal hypoxemia duration in our study. This finding suggests an association between the effect of nocturnal hypoxemia and decreased GMV in OSAS patients.

## Introduction

Obstructive sleep apnea syndrome (OSAS) is a frequent but insufficiently recognized breathing disorder occurring during sleep that affects at least 2–4% of the population aged 30–60 years and up to 20–50% of the elderly population, with a 2:1 men/women ratio in Caucasians (1, 2). It is characterized by recurrent hypoxic episodes during sleep, sleep fragmentation, and changes in sleep architecture, resulting in increased cardiovascular comorbidity, neurocognitive impairment, and mood disorders, as well as excessive daytime sleepiness (3–8).

Several studies have examined whether OSAS also induces morphological brain changes that could underlie those impairments (9–15). To date, results vary widely across structural neuroimaging studies of OSAS, with both positive and negative results. However, among studies that reported structural changes, hippocampal abnormalities are the most consistent finding across different neuroimaging techniques (10, 11, 14, 16, 17).

Voxel-based morphometry (VBM) is a widely used neuroimaging technique that allows non-invasive, region-specific measurement of gray and white matter volume (GMV and WMV) changes in the brain. Previous VBM studies in OSAS patients have reported conflicting results, which cannot be entirely explained by differences in methodologies or disease severity (9–12, 14, 18). The observation that much of the VBM differences in GMV observed in OSAS patients are reversible using continuous positive airway pressure (CPAP) treatment led to the hypothesis that these decreases reflect either neuronal or vasogenic changes, or both (14, 15).

The interest in determining whether OSAS leads to structural brain changes is explained by the fact that some OSAS-related deficits are irreversible, which could be related to OSAS-induced cellular loss. Therefore, determining whether OSAS leads to structural brain changes and if so, mapping their anatomical distribution and extent, as well as understanding their nature, will greatly impact OSAS disease prevention, early diagnosis, and management.

Therefore, the aim of this study was to use VBM analysis to (a) compare GMV and WMV in untreated OSAS patients to those in healthy controls (HC); (b) examine the impact of OSAS-related variables (nocturnal hypoxemia duration and sleep fragmentation index) on GMV and WMV; (c) assess the effects of therapeutic vs. sham CPAP treatment; and (d) examine our findings in light of previously published research.

## Materials and Methods

### Participants

Twenty-seven treatment-naïve male patients with moderate to severe OSAS [apnea–hypopnea index (AHI) ≥ 15] and seven healthy age- and education-matched control subjects (AHI < 5) were recruited from the Stanford Sleep Disorder Clinic through advertisements. All participants were right-handed non-smokers and had regular sleep schedules. Exclusion criteria were other sleep disorders, any neurological or psychiatric disorder, and taking psychotropic medications or medication liable to affect the hemodynamic response (e.g., vasodilators or vasoconstrictors).

After a baseline functional magnetic resonance imaging (fMRI) scan, patients were randomly assigned to either the active (therapeutic, *n* = 14) or sham (subtherapeutic, *n* = 13) nasal CPAP group. A CPAP titration study was conducted on all patients in both groups: the active group was effectively titrated to the appropriate nasal CPAP pressure, and the sham group slept with the subtherapeutic nasal CPAP previously used in sham CPAP studies (19). The sham CPAP device closely simulated the active CPAP device with respect to airflow through the exhalation port and operating noise. A prior study using a functionally similar sham CPAP device revealed that oxygen saturation, end-tidal CO_2_, and mean temperature and humidity measured at the CPAP mask were the same for active and sham CPAP (19), and no significant difference was found in sleep parameters or the number of abnormal respiratory events between the sham CPAP group and a no-treatment group in 10 men with OSAS matched for age and AHI.

Subjects in both groups were treated for 2 months and treatment compliance was monitored using an Encore^®^ Pro SmartCard^®^ system. At the end of the treatment period, the sham CPAP group underwent a second CPAP titration night, were provided with therapeutic CPAP treatment, and left the study.

The study was approved by the Stanford Institutional Review Board (IRB approval No: 5129), and all participants signed an informed consent form. This study was a research fMRI sub-study conducted at one center of a multi-centric clinical trial (APPLES), including five centers and 1200 participants randomized into CPAP and sham CPAP groups (ID No: NCT00051363).

### Overnight sleep studies

Overnight polysomnography (PSG) recordings were performed on all participants at baseline. In OSAS patients, PSG recordings confirmed the moderate to severe OSAS diagnosis (AHI ≥ 15). In healthy volunteers, PSG recordings confirmed the absence of sleep disorders, including OSAS (AHI < 5). PSG recordings included electroencephalography, electrooculogram, electrocardiogram, electromyogram for chin and legs, nasal air flow with nasal cannula, abdominal and thoracic respiratory belts, and pulse oximetry. Measures were scored according to AASM criteria (20). PSG recordings were performed using the CleveMed ambulatory Crystal Monitor^®^or Sandman in-house software.

### Magnetic resonance imaging

All participants underwent an fMRI scan at baseline and after 2 months of either sham or therapeutic CPAP treatment. Imaging data were obtained with a 3.0-T GE scanner (Milwaukee, WI, USA) using a custom quadrature birdcage head coil. Head movement was minimized with foam padding. High-resolution T1-weighted images (TR = 3000 ms, TE = 68 ms, flip angle = 11°, FOV = 25 cm, matrix = 256 × 256, 124 axial slices, slice thickness = 1.2 mm) were collected on each participant using an IR-prep FSPGR sequence for T1 contrast.

### Voxel-based morphometry and statistical analyses

Imaging data were preprocessed and analyzed using SPM8 software (Wellcome Department of Cognitive Neurology, London, UK)^1^ and the VBM8 Toolbox^2^ with default parameters in MatLab 7.9.0. Preprocessing steps included tissue segmentation, high-dimensional DARTEL normalization of modulated data by the non-linear components derived from the normalization matrix (modulated GMV and WMV), and spatial smoothing with a Gaussian kernel of 8 mm full width at half maximum (FWHM).

Untreated OSAS patients were compared to HC at baseline on sleep and treatment compliance variables using one-way ANOVA and two-sample *t*-test. Brain morphology was analyzed with a one-way ANOVA for baseline comparisons between the two groups of untreated OSAS patients and HC. Treatment effects were explored with a flexible factorial model in 21 OSAS patients (11 sham CPAP and 10 therapeutic CPAP), as six patients were excluded for low treatment compliance ( <50% of treatment days at >4 h of CPAP use). The effects of sleep fragmentation (measured with AHI) and nocturnal hypoxemia duration (in minutes) were assessed by multiple regression analysis in untreated OSAS patients.

## Results

At baseline, untreated OSAS patients were equivalent on all sleep and breathing variables. Patients were then randomly assigned to either treatment group. Both OSAS treatment groups demonstrated similar CPAP compliance (see Table 1).

**Table 1:** **Baseline demographics, sleep, and treatment compliance variables for patients with obstructive sleep apnea syndrome (OSAS) treated with sham continuous positive airway pressure (CPAP) device or active CPAP device**.

	Controls	Sham CPAP	Active CPAP	*p*-Value
	*n* = 7	*n* = 13	*n* = 14	
Age (years)	41.4 ± 3.1	44.0 ± 2.0	42.9 ± 2.2	0.78^a^; 0.48^b^; 0.71^c^; 0.70^d^
BMI (kg/m^2^)	24.1 ± 1.2	26.1 ± 0.6	28.7 ± 1.1	**0.01^a^**; 0.12^b^; **0.02^c^**; **0.05^d^**
Total sleep time (min)	349.8 ± 53.5	376.6 ± 11.7	378.7 ± 19.4	0.74^a^; 0.66^b^; 0.53^c^; 0.93^d^
Stage 1 TST (%)	4.1 ± 0.7	11.3 ± 1.8	10.7 ± 2.2	0.20^a^; **0.04^b^**; **0.01^c^**; 0.84^d^
Stage 2 TST (%)	61.8 ± 6.9	61.6 ± 2.5	66.3 ± 3.1	0.50^a^; 0.97^b^; 0.52^c^; 0.25^d^
Stage 3 TST (%)	5.1 ± 1.8	5.9 ± 1.5	4.2 ± 1.2	0.64^a^; 0.76^b^; 0.73^c^; 0.36^d^
Stage 4 TST (%)	0.7 ± 0.4	1.5 ± 0.8	2.1 ± 1.2	0.75^a^; 0.56^b^; 0.53^c^; 0.68^d^
REM TST (%)	23.4 ± 6.1	19.6 ± 1.1	16.7 ± 1.5	**0.01^a^**; 0.25^b^; **0.01^c^**; 0.14^d^
AHI	2.1 ± 0.8	34.0 ± 4.8	43.4 ± 5.9	**0.003^a^**; **<0.001^b^**; **0.002^c^**; 0.23^d^
Sleep efficiency (%)	84.8 ± 5.4	83.4 ± 2.1	82.5 ± 3.6	0.93^a^; 0.78^b^; 0.76^c^; 0.84^d^
Average SPO_2_ (%) total	104.7 ± 7.5	95.4 ± 0.3	93.7 ± 1.0	**0.01^a^**; 0.30^b^; 0.24^c^; 0.11^d^
Number days range	N/A	72.1 ± 5.1	78.3 ± 5.7	0.43^d^
Average usage – all days (h/night)	N/A	4.7 ± 0.5	4.4 ± 0.4	0.57^d^
Percent days >4 h – all days	N/A	67.1 ± 8.5	62.0 ± 6.9	0.64^d^

*^a^Data presented as mean ± SEM. Statistical comparison of the three groups was done with a one-way ANOVA, followed by two-by-two group comparisons with independent *t*-test*.

*^b^Control vs. sham*.

*^c^Control vs. active*.

*^d^Sham vs. active*.

*N/A, not applicable*.

### Gray matter volume

**Figure 1:**
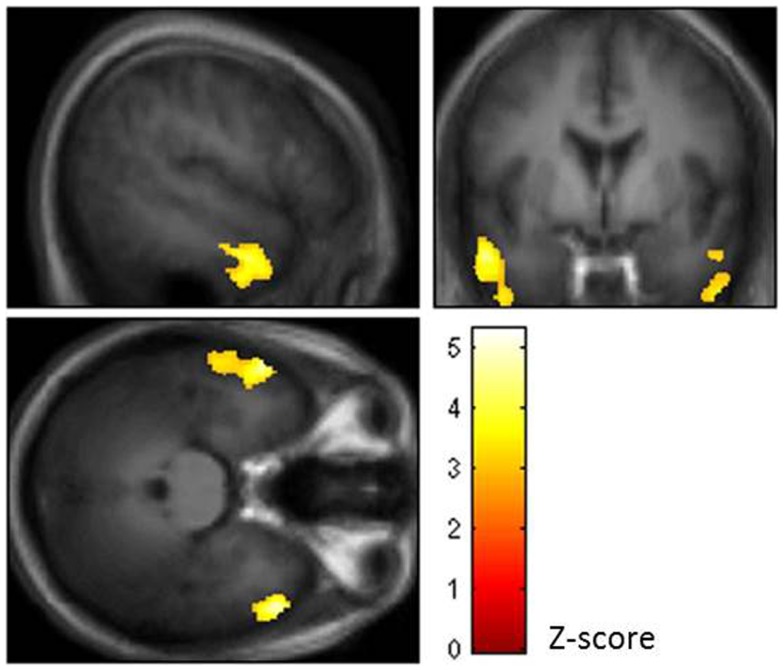
**Brain regions showing negative correlation between nocturnal hypoxemia duration and gray matter volume (GMV) in bilateral lateral temporal regions of the middle and inferior temporal gyri (BA = 20, 21) [topological false discovery rate (FDR) correction for multiple comparisons at cluster level *p* < 0.05, voxel level *p* = 0.005 uncorrected]**.

Using topological false discovery rate (FDR) correction for multiple comparisons at cluster level (*p* < 0.05, voxel level *p* = 0.005 uncorrected), we found no difference in GMV between OSAS patients and HC and no effects of CPAP vs. sham CPAP treatment in OSAS patients. In OSAS patients, we found a significant negative correlation between nocturnal hypoxemia duration ( <90% SaO_2_) and GMV in bilateral lateral temporal regions [middle and inferior temporal gyri: Brodmann Area (BA) = 20, 21] (see Figure 1).

Using a voxel-wise *p* < 0.001 threshold uncorrected for multiple comparisons with a 20-voxel extent threshold for exploratory analysis, we found significantly higher GMV in HC compared to OSAS patients in the left middle frontal gyrus (BA = 46). Significantly higher GMV was found in untreated OSAS patients compared to HC in the right precuneus (BA = 7) and fusiform/lingual gyri (BA = 18) (see Table 2).

**Table 2:** **Gray matter volume (GMV) analysis results: (A) brain regions showing significant differences in GMV between healthy controls (HC) and obstructive sleep apnea syndrome (OSAS) patients; (B) brain regions showing significant correlation with the apnea–hypopnea index (AHI) or with nocturnal hypoxemia duration in OSAS patients (MNI coord.; 20 vox., uncorrected *p* < 0.001)**.

	Region	Brodmann area	*X*	*Y*	*Z*	*Z*-score	Cluster size
**(A) COMPARISON BETWEEN HC AND OSAS PATIENTS**							
HC > OSAS	L middle frontal gyrus	46	−43	44	9	3.31	52
HC < OSAS	R precuneus	7	18	−52	41	3.74	161
	R occipital fusiform gyrus	18	26	−87	−19	3.56	69
	R occipital lingual gyrus	18	2	−86	−7	3.41	123
**(B) CORRELATIONS IN OSAS PATIENTS**							
AHI positive	L postcentral gyrus/inferior parietal lobe	40	−45	−27	26	3.74	123
AHI negative	L medial frontal gyrus	11	−1	48	−17	4.07	229
	R uncus	36	21	1	−35	3.80	187
	R precuneus	7	18	−71	44	3.78	97
Desaturation positive	L precuneus	7	−17	−60	44	4.70	96
Desaturation negative	R superior temporal gyrus	38	46	15	−33	4.17	602
	L middle temporal gyrus	21	−52	6	−22	3.94	383
	R uncus	36	24	1	−30	3.79	198

A significant positive correlation was found between GMV and AHI in the left inferior parietal lobule (BA = 40). Significant negative correlations were found between GMV and AHI in the right uncus (BA = 36), left gyrus rectus (BA = 11), and left precuneus (BA = 7) (see Table 2).

Four OSAS patients were excluded from the multiple comparison analysis due to incomplete nocturnal saturation data. A significant positive correlation was found in the left precuneus (BA = 7). Significant negative correlations between GMV and nocturnal hypoxemia duration were found in the left middle temporal gyri (BA = 21), right superior temporal gyrus (BA = 38), left inferior temporal gyrus (BA = 20), and uncus (BA = 36) (see Table 2).

No regions showed a significant group × session interaction. Furthermore, no correlations were found between CPAP compliance and GMV in bilateral hippocampal regions of interest (ROIs), and no negative correlations with nocturnal hypoxemia.

### White matter volume

Using a voxel-wise *p* < 0.001 threshold uncorrected for multiple comparisons with a 20-voxel extent threshold for exploratory analysis, we found significantly higher WMV in HC compared to OSAS patients in the right occipital lingual gyrus and the left middle occipital gyrus (see Table 3). Similarly to the GMV analyses, four OSAS patients were excluded from the multiple comparison analysis due to incomplete nocturnal saturation data. Significant positive correlations between WMV and nocturnal hypoxemia duration were found in the left medial frontal gyrus and the right frontal gyrus (see Table 3).

**Table 3:** **White matter volume (WMV) analysis results: (A) brain regions showing significant differences in GMV between healthy controls (HC) and obstructive sleep apnea syndrome (OSAS) patients; (B) brain regions showing significant correlation with nocturnal hypoxemia duration in OSAS patients (MNI coord.; 20 vox., uncorrected *p* < 0.001)**.

	Region	*X*	*Y*	*Z*	*Z*-score	Cluster size
**(A) COMPARISON BETWEEN HC AND OSAS PATIENTS**						
HC > OSAS	R occipital lingual gyrus	20	−50	−3	3.87	351
	L middle occipital gyrus	−23	−85	15	3.54	23
**(B) CORRELATIONS IN OSAS PATIENTS**						
Desaturation positive	L medial frontal gyrus	−7	1	48	3.88	69
	R frontal	20	−10	52	3.87	64

## Discussion

Voxel-based morphometry (VBM) allows voxel-wise comparisons of brain structures by performing statistical analyses of MR images that have undergone standard preprocessing, including gray matter (GM) and white matter (WM) segmentation, normalization to an MNI anatomical template, and spatial smoothing. However, normalization introduces a distortion in the local MR signal due to individual structural variability, resulting in overestimation of a given structure’s signal if it has been stretched, or underestimation if it has been reduced to fit the standard anatomical template. To circumvent this issue, Good et al. (21) proposed an additional data treatment step in which GM voxel values are multiplied by Jacobian determinants (representing deformation parameters obtained after normalization), resulting in modulated data. Modulated (GM or WM *volume*) and unmodulated (GMor WM *concentration*) data yield different information about examined brain structures. Modulated GM or WM volume (GMV or WMV) images allow comparing absolute brain volume, thus inferring the presence of atrophy in a given brain structure, whereas unmodulated GM or WM concentration (GMC or WMC) images allow comparing the ratio of GM or WM to other brain tissue present in a given brain structure (22). However, unmodulated images do not allow inferences about structural volume differences between brain structures indifferent subjects or groups of subjects. Moreover, GMC can be misinterpreted as meaning the concentration of neurons in a given voxel, leading to erroneous interpretations of potential cellular injury. Therefore, to respond to the question of whether the brain of OSAS patients presents global or focal parenchymal atrophy compared to the brain of HC, analyzing GMV and WMV appears more appropriate than analyzing GMC and WMC.

Although VBM studies of OSAS patient brains have obtained highly inconsistent results, GMV analyses show few or no differences between patients and HC. Moreover, the majority of studies reporting GMV differences present results that are uncorrected for multiple comparisons, suggesting that the observed effects are of small magnitude (see Table 4).

**Table 4:** **VBM studies in OSA**.

Reference	VBM specification	OSA patients				Healthy controls				Major findings
		*N*	Age (years)	BMI	AHI	*N*	Age (years)	BMI	AHI	
(10)	*p* < 0.001 uncorrected for multiple comparisons, extent threshold = 350 voxels, patients were not screened for cardiovascular or psychiatric comorbidities	21 Men (2 were left-handed)	49 ± 11	30 ± 4	RDI 38 ± 24 events/h	21 Men (4 were left-handed)	47 ± 11	27 ± 4	N/A	GM-volume loss in patients was reported in the right postcentral gyrus, posterior lateral parietal cortex bilaterally and anterior superior frontal cortex bilaterally, bilateral parahippocampal gyri among others
										No regions had higher gray matter volume in patients and no difference between groups was found for white matter
(11)	*p* < 0.05 corrected for multiple comparisons, patients were not screened for cardiovascular or psychiatric comorbidities	7 Men				7 Men				GM-concentration is lower in patients in left hippocampus
										No further significant GM-concentration differences between groups
										GM-volume was not different between groups
(12)	*p* < 0.001 uncorrected for multiple comparisons (optimized VBM), effects of 6 months of CPAP treatment, two patients and one control subject were excluded from VBM analysis	27 Men	45.7 ± 10.1	33.2 ± 4.7	71.7 ± 17.0	24 Subjects	43.3 ± 9.4	25.3 ± 2.8	5.9 ± 4.7	GM-volume loss in patients was reported in the posterior and mesial temporal lobe bilaterally and the left insular region
										GM-volume increase in patients as compared to healthy controls was reported in the right basal ganglia and less prominently in scattered frontal lobe and parietal lobe areas
(24)	*p* < 0.005 uncorrected for multiple comparisons at voxel level, *p* < 0.05 corrected at cluster level (optimized VBM), patients were not screened for cardiovascular comorbidities	16 Patients (15 M: 1 W)	54.8 ± 5.7	N/A	38.3 ± 14.3	19 Subjects from a brain database in France, although not screened for sleep apnea	55.3 ± 6.7	N/A	N/A	GM-concentration loss in patients in the frontal and temporo–parieto–occipital cortices, the thalamus, some of the basal ganglia and the cerebellar regions, mainly right-lateralized
										No information was provided as to whether OSAS patients also displayed regions of higher GM-volume as compared to healthy controls
(16)	*p* < 0.05 and cluster size > 200 (FDR correction for multiple comparisons), modulated and unmodulated data	36 Men	44.7 ± 6.7	26.0 ± 2.7	52.5 ± 21.7	31 Subjects	44.8 ± 5.4	25.8 ± 3.3	2.8 ± 0.9	GM-volume showed no regional differences between OSAS patients and health controls
										GM-concentration reduction in OSAS patients was found in left gyrus rectus, bilateral superior frontal gyri, left precentral gyrus, bilateral frontomarginal gyri, bilateral anterior cingulate gyri, right insular gyrus, bilateral caudate nuclei, bilateral thalami, bilateral amygdalo-hippocampi, bilateral inferior temporal gyri, and bilateral quadrangular and biventer lobules in the cerebellum
										There was no region with higher GM-concentration in patients than healthy controls
										GM-volume and -concentration showed no correlation with clinical parameters (age, AHI, arousal index, and Epworth sleepiness scale)
(25)	*p* < 0.05 at cluster level (FDR correction for multiple comparisons) (optimized VBM), two different sites of MRI acquisition	60 Patients (57 M: 3 F)	47.3 (44.2–50.3) Mean (95% CI)	32.0 (30.9–33.1)	55.0 (48.3–61.6)	60 Subjects (55 M: 5 F)	46.1 (43.2–49.0)	25.0 (24.1–25.9)	4.1 (3.0–5.1)	GM-volume decreased in patients in the right middle temporal gyrus and cerebellar regions
										No information was provided as to whether OSAS patients also displayed regions of higher GM-volume as compared to healthy controls
(17)	*p* < 0.001 uncorrected for multiple comparisons, *p* < 0.05 cluster-wise family-wise error correction	16 Patients (13 M: 3 F)	55.8 ± 6.7	31.7 ± 4.4	52.5 ± 26.0	14 Subjects (9 M: 5 F)	57.6 ± 5.2	25.5 ± 2.4	N/A	Global cortical GM-volume reduced in patients
										GM-volume decreased in patients in the right hippocampus (*p* < 0.05 PFWE). In addition to the following areas: left hippocampus and some lateral temporal of both hemispheres (*p* < 0.001 uncorrected)
										WM-volume decreased in patients in the right temporal lobe
(14)	*p* < 0.05 cluster-wise family-wise error correction, *p* < 0.005 uncorrected at voxel level, effects of 3 months of CPAP treatment	17 Men	44.0 ± 7.6	31.2 ± 4.4	55.8 ± 19.1	15 Men	42.2 ± 6.6	26.1 ± 2.5	1.6 ± 1.5	GM-volume decreased in patients in the left posterior parietal cortex, right superior frontal gyrus, and left parahippocampal gyrus. A negative correlation with AHI, desaturation duration <90%, and GM-volume in left posterior parietal cortex
										GM-volume showed no increases in patients
										Global cortical GM- and WM-volumes were different between groups
										GM-volume showed no significant reduction in patients following CPAP.
										GM-volume increased in patients in the hippocampal and frontal structures following CPAP, which was also correlated with AHI and desaturation duration in the right entorhinal cortex and left subiculum

The first VBM-based study in OSAS patients was published by Macey et al. (10) (1.5 T) in 21 patients with a mean respiratory disturbance index (RDI) of 38 ± 24 events/h. The analysis was performed on unmodulated data (GMC), and patients were not screened for cardiovascular or psychiatric comorbidities. Results were reported for *p* = 0.001 uncorrected for multiple comparisons at a 350-voxel extent.

Higher GM signals were reported in OSAS patients compared to HC in the right postcentral gyrus, posterior lateral parietal and anterior superior frontal cortex bilaterally, and bilateral parahippocampal gyri, among others. No regions showed higher GM signals in OSAS patients compared to HC, and no differences in WM were found.

Morrell et al. (11) (1.5 T) also examined unmodulated data (GMC) in seven male OSAS patients with a mean AHI of 28 events/h and found lower GMC in OSAS patients compared to HC in the left hippocampal region of interest (*p* = 0.004, corrected for multiple comparisons, small volume correction). They found no differences between HC and patients on whole brain comparison, *p* = 0.05 corrected for multiple comparisons. The same research group found similar findings in 22 patients in another study, with decreased GMC in bilateral posterior hippocampal regions (*p* = 0.001, ROI analysis, uncorrected for multiple comparisons) (23).

The first study to use modulated VBM data (GMV) and to investigate the effects of 6 months of CPAP treatment was performed by O’Donoghue et al. (12) (3 T) in 27 patients with severe OSAS (AHI: 74 events/h) without comorbidities. Using a *p* threshold of 0.001 uncorrected for multiple comparisons, they observed scattered areas of GM deficit in patients compared to control subjects, including the posterior and mesial temporal lobe bilaterally and the left insular region. A reverse contrast showed increased GMV in OSAS patients compared to HC in the right basal ganglia, and less prominently in scattered frontal lobe and parietal lobe areas. However, using multiple comparison correction, they found no differences between OSAS patients and HC, and no changes in OSAS patients after 6 months of CPAP treatment.

Yaouhi et al. (24) (1.5 T) examined brain structure in 16 newly diagnosed OSAS patients (AHI: 38 events/h) using both PET and VBM (unmodulated VBM data). Voxel-wise GMC analysis at *p* = 0.005 uncorrected for multiple comparisons (*p* = 0.05 corrected at cluster level) revealed scattered sites of GMC loss in OSAS patients in the frontal and temporo–parieto–occipital cortices, the thalamus, some of the basal ganglia, and the cerebellar regions. No results were provided on the opposite contrast, that is, whether OSAS patients also showed regions of higher GMC compared to HC. Macey et al. (10) found similar results in subjects with cardiovascular comorbidities.

A study by Joo et al. (16) (1.5 T) examined both modulated and unmodulated data from 36 male OSAS patients without cardiovascular, neurological, or psychiatric comorbidities (AHI: 52.5 events/h). Using FDR correction for multiple comparisons (*p* = 0.05 and cluster size >200), they found no differences in GMV between OSAS patients and HC, and no brain regions with significant correlations between clinical parameters (age, AHI, arousal index, and Epworth Sleepiness Scale) and GMV. However, GMC was significantly reduced in OSAS patients compared to HC in the bilateral superior frontal gyri, left gyrus rectus, bilateral frontomarginal gyri, bilateral anterior cingulate gyri, right anterior insular gyrus, bilateral caudate nuclei, bilateral thalami, bilateral amygdalohippocampal gyri, bilateral inferior temporal gyri, and bilateral cerebellar cortices. No region showed higher GMC in patients compared to HC.

Canessa et al. (14) (3 T) conducted another VBM study to examine the effects of CPAP treatment on GMV in OSAS patients compared to HC. They studied 17 treatment-naïve OSAS patients (AHI: 55.8 events/h) without associated comorbidities. Using a family-wise error (FWE) cluster-level correction for multiple comparisons (*p* = 0.05, voxel level *p* = 0.005), they found significantly reduced GMV in pretreatment patients compared with control subjects in the left posterior parietal cortex and right superior frontal gyrus. No region showed higher GMV in patients with OSAS compared to HC. A significant negative correlation was found between AHI and time of nocturnal desaturation below 90% SaO_2_ and GMV in the left posterior parietal cortex. Furthermore, specifically in the hippocampal region of interest with an uncorrected *p* = 0.005 at voxel level, they found decreased GMV in OSAS patients compared to HC. After 3 months of CPAP treatment, specific hippocampal (left subiculum and bilateral entorhinal cortex) and frontal (superior and middle frontal gyri and medial orbitofrontal cortex) clusters showed increased GMV (FDR corrected at cluster level, *p* = 0.05). Moreover, after CPAP treatment, overall GMV was significantly increased in patients with OSAS, despite no significant increase in total intracranial volume.

The largest VBM study to date, recently published by Morrell et al. (25), investigated GMV in 60 OSAS patients (AHI: 50 events/h) without comorbidities examined at two different sites (1.5 and 3 T). Using topological FDR correction for multiple comparisons (*p* = 0.05, cluster level), they found lower GMV in the right middle temporal gyrus and cerebellar regions in patients with OSAS compared to HC. No information was provided as to whether results were provided on higher GMC in OSAS patients also displayed regions of higher GM-concentration compared to HC.

Torelli et al. (17) examined GMV in 16 newly diagnosed OSAS patients (AHI: 63 events/h) without major cardiovascular disorders (3 T). They found reduced cortical GMV in OSAS patients compared to controls (*p* = 0.01, multiple comparison correction data not provided), as well as a region of decreased GMV in the right hippocampus when applying cluster-wise FWE correction for multiple comparisons (*p* = 0.05).

The above studies indicate that decreased GMV in OSAS patients compared to HC, when applying a statistical correction for multiple comparisons, were found only in the right temporal cortex (25) and the left posterior parietal and right superior frontal corci (14). Increased GMV after 3 months of CPAP treatment were found in the hippocampal and frontal regions, differing from brain regions that showed decreased GMV compared to HC at baseline (14).

The results of our study are in line with previous observations, as we found no GMV or WMV differences between 27 treatment-naïve OSAS patients and HC when data were corrected for multiple comparison. Using a more lenient threshold (voxel-wise *p* = 0.001 uncorrected, 20-voxel extent threshold), we found small GMV decreases in the left frontal cortex, but also increases in the right parietal and occipital regions. Given that our subjects had less severe disease (AHI, nocturnal hypoxemia) than subjects in previous studies, our results extend previous observations to a more moderate range of OSAS and support the finding that GMV changes are not prevalent in the brain of OSAS patients.

Interestingly, compared to the findings of Morrell et al., we found a significant negative correlation (*p* = 0.05, FDR corrected at cluster level) between nocturnal hypoxemia duration and GMV in the same region of the temporal lobe as their finding of decreased GMV in OSAS patients compared to HC. This suggests an association between the effect of nocturnal hypoxemia and decreased GMV in OSAS patients compared to HC. We did not expect the lateral temporal regions to show a negative correlation with hypoxemia, whereas the hippocampal regions did not. However, as Morrell et al. noted, this area is susceptible to hypoxic damage in an animal model of OSAS (25). Neurons in the dentate gyrus of the hippocampal formation have been shown to maintain their ability to regenerate throughout adult life, and this neurogenesis can be inhibited by sleep fragmentation or deprivation (26). The fact that hippocampal neurons can regenerate even though they are sensitive to oxidative injury, but that the neurogenesis can be inhibited by insufficient sleep, could explain the inconsistent VBM findings in the hippocampus of OSAS patients.

The fact that we found no difference between patients and controls, or between treatment groups while finding a significant correlation with nocturnal hypoxemia in the temporal lobe GMV may appear contradictory, but in reality reflects two different aspects of OSAS effects. On one hand, it indicates that OSAS-related morphological changes, if present, are of small magnitude. On the other, it demonstrates that between AHI and nocturnal hypoxemia duration, the latter is the OSAS-related factor that has the most consistent effect on GMV, particularly in the temporal lobes. Lateral temporal regions that showed correlation with hypoxemia in our study and differences in GMV in Morrell et al.’s work (11, 23, 25) are involved in various associative processes, particularly memory processing. However, in order to better understand the relationship between these cortical areas and cognitive deficits in OSAS patients, further larger patient sample studies that simultaneously measure both GMV and cognitive impairment are needed. Moreover, more severe hypoxemia is often seen in overweight or obese patients with OSAS. A larger group of patients would allow investigation of findings in normal weight and overweight OSAS patients and of the role of obesity by dissociating OSAS from obesity.

It is unclear why disease severity appears to correlate poorly with GMV changes in OSAS patients. One possible explanation is that factors other than disease severity or vascular comorbidity could be at play. In particular, Alchanatis et al. (27) showed that high intelligence (measured by IQ) has a protective effect on the cognitive function of patients with OSAS. No VBM studies to date have included a measure of education level (which is a surrogate for many personal, socioeconomic, and cognitive variables) in their analysis, and further studies are needed to determine whether patients’ education level can explain the discrepancies observed in structural brain studies.

Another question that remains unanswered is which pathophysiological processes are represented by changes in VBM, GM, or WM signals. It has been postulated that decreased GMV represents cellular loss, as seen in local and diffuse brain atrophy. However, in view of the fact that both decreased and increased signals have been observed in OSAS patients, Canessa et al. (14) proposed that changes in VBM signals may be less specific, and may represent neuronal or vascular processes, or both. Future studies could aim to elucidate this question by combining VBM with perfusion and water-sensitive imaging.

In conclusion, in terms of VBM changes in GMV, there is little difference between OSAS patients and HC. The largest VBM study to date points to structural changes in the lateral aspect of the temporal lobe, which also demonstrated a significant negative correlation with nocturnal hypoxemia duration in our study. Further research is needed to elucidate the potential protective role of cognitive reserve and to distinguish the neuronal and vascular glial contributions to VBM-measured GMV signal changes.

## Conflict of Interest Statement

The authors declare that the research was conducted in the absence of any commercial or financial relationships that could be construed as a potential conflict of interest.

## Footnotes

^1^http://www.fil.ion.ucl.ac.uk/spm

^2^http://dbm.neuro.uni-jena.de/vbm/
